# Eleven years of data on the Jefferson Scale of Empathy – medical student version: Japanese norm data and tentative cutoff scores

**DOI:** 10.1186/s12909-022-03977-5

**Published:** 2023-02-02

**Authors:** Hitomi U. Kataoka, Akiko Tokinobu, Chikako Fujii, Mayu Watanabe, Mikako Obika

**Affiliations:** 1 Center for Diversity and Inclusion, Okayama University Hospital, 2-5-1, Shikata-Cho, Kita-Ku, Okayama-Shi, Okayama, 700-8558 Japan; 2 Division of Kidney, Diabetes and Endocrine Diseases, Okayama University Hospital, 2-5-1, Shikata-Cho, Kita-Ku, Okayama-Shi, Okayama, 700-8558 Japan; 3 Department of General Medicine, Faculty of Medicine, Dentistry and Pharmaceutical Sciences, Okayama University, 2-5-1, Shikata-Cho, Kita-Ku, Okayama-Shi, Okayama, 700-8558 Japan

**Keywords:** Jefferson Scale of Empathy, Norm data, Cutoff scores, Medical students, Empathy

## Abstract

**Background:**

More and more studies investigate medical students’ empathy using the Jefferson Scale of Empathy (JSE). However, no norm data or cutoff scores of the JSE for Japanese medical students are available. This study therefore explored Japanese norm data and tentative cutoff scores for the Japanese translation of the JSE-medical student version (JSE-S) using 11 years of data obtained from matriculants from a medical school in Japan.

**Methods:**

Participants were 1,216 students (836 men and 380 women) who matriculated at a medical school in Japan from 2011 to 2021. The JSE-S questionnaire was administered to participants prior to the start of the program. Data were summarized using descriptive statistics and statistical tests were performed to show the norm data and tentative cutoff scores for male and female students separately.

**Results:**

The score distributions of the JSE-S were moderately skewed and leptokurtic for the entire sample, with indices -0.75 and 4.78, respectively. The mean score (standard deviation) for all participants was 110.8 (11.8). Women had a significantly higher mean score (112.6) than men (110.0; *p* < 0.01). The effect size estimate of gender difference was 0.22, indicating a small effect size. The low and high cutoff scores for men were ≤ 91 and ≥ 126, respectively, and the corresponding scores for women were ≤ 97 and ≥ 128, respectively.

**Conclusions:**

This study provides JSE-S norm data and tentative cutoff scores for Japanese medical school matriculants, which would be helpful in identifying those who may need further training to enhance their empathy.

## Background

Empathy can be described as the competence of a physician to understand their patient’s situation, perspective, and feelings; to communicate this understanding and confirm its accuracy; and to act on that understanding with the patient in a helpful (therapeutic) way [1]. Empathy has been described as an essential component of overall physician competence [2]. Previous studies demonstrate the effectiveness of physician empathy in relation to positive patient outcomes among diabetic patients [3, 4]. Moreover, studies also show increased patient enablement [5] and patient satisfaction [6]. Medical students’ empathy has also been reported to be associated with their clinical competence [7, 8].

To date, numerous studies have investigated the level of empathy among medical students in various countries [9–17]. Although medical students’ empathy may increase or decrease during their education, their levels of empathy in the first year of medical school have shown a tendency to be higher in Western countries (e.g., the United States [US] and the United Kingdom) than in Asian countries (e.g., India, South Korea, and Japan) [18]. The determinants of why student empathy varies according to geographical regions remain unknown. To explore the factors of geographical differences in empathy among medical students, internationally comparable norm data from different countries or regions are required.

Norm data and cutoff scores of first-year medical students’ empathy in the US [19] and Spain [20] have already been reported using the Jefferson Scale of Empathy (JSE). The JSE is a validated psychometric instrument specifically developed to measure empathy in the context of patient care among healthcare professional practitioners and students. It has been widely used for different healthcare professional practitioners and students, translated into 56 languages/dialects, and used in at least 85 different countries [21]. A detailed description of the JSE is available elsewhere [22, 23].

## Objectives

Several studies on empathy among Japanese medical students have been conducted using the Japanese translation of the JSE [17, 24–26]. However, no norm data or cutoff scores for the Japanese JSE among Japanese medical students have been recorded. If norm data and cutoff scores are available, we can classify the students as having lower, moderate, or higher levels of empathy, which allows us to investigate the underlying factors associated with empathy level. In turn, this may help us to identify appropriate intervention measures to increase students’ empathy according to its original level. Therefore, to address this necessity, we explored Japanese norm data and tentative cutoff scores of the JSE, using 11 years of data, from 2011 to 2021, obtained from matriculants enrolled at a medical school in Japan.

## Methods

### Study design and participants

This descriptive cross-sectional study used data from the medical school of Okayama University in Japan, from April 2011 to April 2021.

Study participants included 1,216 students (836 men and 380 women) who matriculated at the medical school of Okayama University in Japan from the academic years of 2011 to 2021, and who responded to the survey at the beginning of their medical school education (representing a 97.5% response rate).

### Instrument

There are currently three versions of the JSE: (1) An “HP” version for physicians and practitioners of all healthcare professions, (2) an “S” version for medical students, and (3) an “HPS” version for students in all healthcare professions other than medicine. These three versions are all very similar in context, with only slight differences in a few words used to adjust the instrument for its target population [19, 27]. In the present study, we used the Japanese translation of the “S” version (JSE-S). The details of the JSE-S have been described previously [11, 19]. Moreover, the validity and reliability of the Japanese translation of the JSE-S have been confirmed [17].

The JSE questionnaire is comprised of 20 question items. Each item is answered on a 7-point Likert-type scale from 7 = “strongly agree” to 1 = “strongly disagree.” The S-version, for example, includes questions such as “Physicians’ understanding of the emotional status of their patients, as well as that of their families, is one important component of the physician–patient relationship.”

### Procedure

Prior to the first class of the medical program, which is provided just after entry into medical school each year, we explained the study to the students, orally and in writing, and asked them to participate. We ensured that they were aware that participation was voluntary, that their responses would be kept strictly confidential, that their answers would not affect their academic record, and that the data might be used as aggregated data for statistical analysis. A hard copy of the JSE-S questionnaire was distributed to each student entering classes of 2011–2019; for the 2020 and 2021 classes, it was administered online. Students who consented to participate in the study filled out and submitted the questionnaire.

This study protocol was approved by the ethics committee of Okayama University Graduate School of Medicine, Dentistry and Pharmaceutical Sciences and Okayama University hospital (Approval No. 826 and Ken 2207–024).

### Statistical analysis

Submitted questionnaires with missing information on more than four items (of the 20) were considered incomplete and excluded from the analysis. If four or fewer items were missing, each missing value was replaced with the mean score calculated from the completed items [22, 28]. Previous studies have required a minimum JSE score of 50 [22], so questionnaires with a total score of less than 50 were marked as invalid and excluded from this study. However, this was only a negligible amount, as only two participants scored less than 50 on our questionnaire (< 0.3%).

For comparison with previous studies, we summarized the data using descriptive statistics and performed the following statistical tests [19]. First, we summarized the data of the JSE-S scores with descriptive statistics, which included frequency, mean, standard deviation (SD), median, range, skewness, and kurtosis indices across matriculation years. We also calculated the Cronbach’s α coefficient—an index of internal consistency and reliability of the JSE-S across matriculation years. Second, we performed an analysis of variance (ANOVA) to examine the differences in the mean JSE-S scores across matriculation years. Third, we used the *χ*^2^ test to determine whether the distribution of gender among the participants was similar across matriculation years. Fourth, we examined the difference in the mean JSE-S scores between male and female students using a two-tailed Student’s *t*-test. We also calculated Cohen’s *d* as an estimate of effect size. The effect size *d* values of 0.8, 0.5, and 0.2 were considered large, medium, and small effect sizes, respectively [29].

Finally, we determined cutoff scores to identify low or high scores. Low and high scores were defined as points below the 7th percentile and above the 93rd percentile, respectively, as indicated by a previous study [19]. As gender differences in the JSE-S have been reported [7, 25, 30], we determined the cutoff scores separately for men and women, from their respective score distributions.

Two-sided *p*-values below 0.05 were considered statistically significant. The data were analyzed using Stata SE 17.0 for Windows (Stata Corp, College Station, Texas, USA).

## Results

The participants included in the analyses were 1,216 students (836 men and 380 women), and the overall response rate was 97.5% (men 97.1% and women 98.4%).

Descriptive statistics, including the frequency, mean, SD, median, score range, skewness, and kurtosis indices of the JSE-S by matriculation year, are presented in Table 1. The mean score (SD) for all participants was 110.8 (11.8); mean scores varied from a low of 108.4 (13.8) for the matriculants of 2014 to a high of 112.5 (11.6) for the matriculants of 2011. The ANOVA revealed no statistically significant differences in the mean scores across matriculation years (*p* = 0.16). The skewness index was negative for the entire sample (-0.75) and for each matriculation year (range: -2.17 [for matriculants of 2021] to -0.17 [for matriculants of 2018]). The kurtosis for the entire sample was 4.78 (range: 2.39 [for matriculants of 2020] to 9.54 [for matriculants of 2021]). The Cronbach’s α coefficient for the entire sample was 0.81 (range: 0.75 [for matriculants of 2012, 2017, and 2018] to 0.87 [for matriculants of 2021]).

**Table 1:** The frequency, mean, standard deviation, median, range, skewness and kurtosis indices, and reliability (Cronbach’s α) coefficients of the JSE-S by matriculation year and the result of an ANOVA

Matriculation year	Students, n	Mean (SD)	Median	Range	Skewness	Kurtosis	Cronbach's α
2011	113	112.5 (11.6)	113	59—134	-1.10	6.31	0.78
2012	114	110.8 (10.7)	111.5	74—134	-0.26	3.34	0.75
2013	113	108.9 (12.8)	108	58—134	-0.46	4.01	0.82
2014	112	108.4 (13.8)	107.5	59—138	-0.34	3.06	0.86
2015	113	109.9 (12.0)	110	73—136	-0.42	2.89	0.83
2016	115	111.4 (11.9)	112	60—133	-0.98	5.35	0.79
2017	114	110.5 (10.4)	111.5	86—134	-0.22	2.65	0.75
2018	112	112.2 (9.4)	112	87—136	-0.17	2.63	0.75
2019	100	110.3 (10.8)	110	80—129	-0.31	2.46	0.79
2020	109	112.3 (11.3)	113	85—136	-0.20	2.39	0.78
2021	101	111.4 (14.2)	113	50—134	-2.17	9.54	0.87
Total	1,216	110.8 (11.8)	111	50—138	-0.75	4.78	0.81

*JSE-S* Jefferson Scale of Empathy-medical student version, *ANOVA* Analysis of variance, SD Standard deviation

ANOVA: *F*_10, 1,205_ = 1.43 (*p* = 0.16, nonsignificant)

Table 2 shows the distribution of the participants by matriculation year and gender as well as the gender differences in the JSE-S mean scores by matriculation year. The proportion of men was higher across all matriculation years. However, the gender composition differed across matriculation years (*p* = 0.007). Women consistently tended to obtain higher scores than men, except in the matriculation year of 2012; however, there were no significant differences in the mean score between men and women in any of the matriculation years. For all participants, women had a significantly higher mean score (112.6) than men (110.0), owing to the large sample size (*p* = 0.0004). The effect size estimates of the mean score differences between men and women varied according to matriculation year (range: -0.10 [for matriculants of 2012] to 0.42 [for matriculants of 2013]). For all participants, the effect size estimate of gender difference was 0.22, indicating a small effect size.

**Table 2:** Gender distribution and gender differences on the JSE-S scores by matriculation year

Matriculation year	Men		Women		t	*p*-value	Effect size^a^
	n	Mean (SD)	n	Mean (SD)			
2011	70	112.0 (11.0)	43	113.1 (12.6)	0.48	0.634	0.09
2012	81	111.1 (10.7)	33	110.0 (10.9)	-0.49	0.622	-0.10
2013	86	107.7 (13.1)	27	113.0 (10.9)	1.91	0.058	0.42
2014	78	107.2 (14.4)	34	111.2 (12.3)	1.43	0.156	0.29
2015	88	109.2 (12.6)	25	112.6 (9.7)	1.24	0.217	0.28
2016	83	110.4 (12.2)	32	113.8 (10.6)	1.40	0.165	0.29
2017	86	110.2 (10.2)	28	111.5 (11.1)	0.58	0.563	0.13
2018	68	111.8 (9.6)	44	112.8 (9.1)	0.55	0.581	0.11
2019	70	109.4 (10.8)	30	112.3 (10.5)	1.22	0.226	0.27
2020	64	110.8 (11.7)	45	114.3 (10.4)	1.62	0.109	0.31
2021	62	110.7 (15.2)	39	112.6 (12.8)	0.64	0.523	0.13
Total	836	110.0 (12.1)	380	112.6 (11.0)	3.57	0.0004	0.22

Gender distribution across matriculation years: *χ*^2^_10_ = 24.0819 (*p* = 0.007, significant)

*JSE-S* Jefferson Scale of Empathy-medical student version, *SD* Standard deviation

^a^ Cohen’s *d*

The frequency distributions of JSE-S scores and percentile ranks for men, women, and the full sample are shown in Table 3. The mean (SD) and median for all participants were 110.8 (11.8) and 111, respectively. The low and high cutoff scores for men were ≤ 91 and ≥ 126, respectively, and the corresponding scores for women were ≤ 97 and ≥ 128, respectively.

**Table 3:** Frequency and percentage distributions and descriptive statistics of scores on the JSE-S by gender

Score interval	Men (*n* = 836)			Women (*n* = 380)			Total (*n* = 1,216)		
	frequency	cumulative frequency	percentile ranks	frequency	cumulative frequency	percentile ranks	frequency	Cumulative frequency	percentile ranks
≤ 80	11	11	1%	3	3	< 1%	14	14	< 1%
81 ー 85	9	20	2%	1	4	1%	10	24	< 2%
86 ー 90	26	46	3—5%	5	9	2%	31	55	2—4%
91 ー 95	48	94	6—11%	10	19	3—5%	58	113	5—9%
96 ー 100	79	173	12—20%	23	42	6—11%	102	215	10—17%
101 ー 105	109	282	21—33%	55	97	12—25%	164	379	18—31%
106 ー 110	135	417	34—49%	67	164	26—43%	202	581	32—47%
111 ー 115	112	529	50—63%	64	228	44—60%	176	757	48—62%
116 ー 120	139	668	64—79%	51	279	61—73%	190	947	63—77%
121 ー 125	101	769	80—91%	58	337	74—88%	159	1106	78—90%
126 ー 130	57	826	92—98%	30	367	89—96%	87	1193	91—98%
131 ー 135	6	832	99%	13	380	97—100%	19	1212	99%
> 135	4	836	100%	0	380	100%	4	1216	100%
Mean score^a^	110.0			112.6			110.8		
SD	12.1			11.0			11.8		
Median score	111			113			111		
Possible range	20—140			20—140			20—140		
Actual range	50—138			53—135			50—138		

JSE-S, Jefferson Scale of Empathy-medical student version

^a^ *t*_1,216_ = 3.6 (*p* = 0.0004 for testing the null hypothesis that JSE mean scores for men and women are not different)

## Discussions

The present study aimed to determine the norm data and tentative cutoff scores for the JSE-S instrument among Japanese medical students, using data of 11 years obtained from a medical school in Japan. The reported norm data have potential implications for medical education in comparing the individual JSE-S scores to determine their relative percentile ranks. For example, the JSE-S score of a male medical student that falls between 121 and 125 would place him in the top 80‒91 percentile relative to the score of another male medical student whose JSE-S score falls between 111 and 115, thus placing him in the 50‒63 percentile.

### Comparison with previous studies

Here, we discuss our findings on norm data and tentative cutoff scores for the JSE-S among medical school matriculants in Japan, compared to previous studies conducted in the US (*N* = 2,637) [19] and Spain (*N* = 893) [20] for first-year medical students. First, we compared our findings with those of the American study, incorporating 11 years’ data, from 2002 to 2012 [19]. The mean JSE-S scores for this study were higher than those of Japan by approximately two and four points for men and women, respectively. Several factors may contribute to this difference. First, the age at entry to medical school is higher in the US. According to a study reporting the characteristics of matriculants in medical schools in the US, more than 98% of matriculants between 2001 and 2015 were 21 years old or older at the time of entry [31]. The mean age of the US study’s participants was 23.4 years old [19]. In Japan in recent years, approximately 85% of matriculants in medical schools are younger than 21 years at the time of matriculation [32]. Most Japanese students enter medical school immediately or within a few years of completing high school.

Second, matriculants in the US have a broad background in their undergraduate majors, including humanities, arts, and social sciences [33, 34]. A previous study demonstrates that first-year osteopathic medical students in the US who had majored in “Social and Behavioral Sciences” and “Arts and Humanities” had higher mean scores of JSE-S than those with a background in “Chemical and Physical Sciences” [30]. In addition, they have more experience before entering medical school, such as following another career, engaging in family obligations, or international travel, living, and working experiences [35, 36]. Most matriculants in Japan, however, enter medical school directly from high school, with chemistry and physical sciences courses, with the exception of those who may have failed their entrance exam at their first attempt. It is possible that first-year Japanese students had not yet cultivated empathy, having just been freed from the severe effort of passing the highly competitive entrance examination. Thus, matriculants in the US were likely to be more mature personally, with more experiences and exposure to situations that would foster empathy. This may be the reason for the finding of the baseline mean score of the JSE-S being higher among American students than among Japanese students.

The type of survey administration—that is, hard copy questionnaire for the matriculants of 2011–2019 or online questionnaire for the matriculants of 2020–2021—did not affect JSE-S scores (data not shown). This result is consistent with that of the US [19].

Next, we compared our findings with those of a Spanish study conducted in eight medical schools in Madrid in 2019 [20]. The mean JSE scores in Spain were higher than those in Japan by approximately seven and eight points for men and women, respectively. The participants in the Spanish study were first-year medical students who had not yet been in contact with patients, and the mean age when the survey was conducted was 18.9 years; this is similar to our study. Differences in response rates and in the JSE version used for the survey could be factors causing the difference in mean scores between Spain and Japan. The response rates of Spanish and Japanese studies were 59.7 and 97.5%, respectively. While most Japanese students responded to the questionnaire, Spanish students who had relatively high empathy might have selectively responded, leading to higher mean scores among Spanish participants.

The JSE version used in the Spanish study was the HP version for physicians and practitioners of all healthcare professions, instead of the S-version for medical students. The JSE was originally developed to measure medical students’ orientation or attitudes toward physician empathy in patient-care situations; that is, the S-version. Thereafter, the HP version was developed to measure empathy among practicing physicians and other healthcare professionals. Although the two versions are very similar in context, the wording of the HP version was modified slightly for some items, to make them more relevant to caregivers’ empathic behavior, than to empathic orientation or attitudes among physicians. For example, the following appeared in the S-version: “Because people are different, it is difficult for physicians to see things from their patients’ perspectives.” In the HP version, this item was revised to read as follows: “Because people are different, it is difficult for me to see things from my patients’ perspectives” [37]. It is possible that the respondents to the S-version were scored more strictly, as this version refers to the empathy of a physician in general, not the empathy of the respondents themselves. This might have contributed to the lower mean score of the Japanese students who used the JSE-S version, compared with the Spanish students who used the HP version.

Cultural traits may also be important factors that affect empathy. A previous study investigating the difference in mean JSE-S scores in relation to race and ethnicity among American osteopathic medical students showed that Asian students had lower mean scores than White/Caucasian or Hispanic/Latino/Spanish students [30]. In general, Japanese people tend to communicate with others in a manner that is calm, ambiguous, humble, and censoring of themselves [38]. It is likely that many Japanese patients hesitate to express their personal feelings or emotions to others, including medical staff. These culture-specific characteristics might have resulted in differences in empathy scores between the Japanese and American or Spanish students. These differences may also originate from the differences between concepts of medical professionalism in Japanese and Western culture. One article [39] suggests that the *Bushido*, a Japanese code of personal conduct originating from ancient samurai warriors, may influence the behaviors of modern Japanese doctors. The *Bushido* contains concepts that differ from the physician charters used in Western medical societies, such as autonomy of the individual, gender roles, and ethical conception. However, these assumptions require further investigation in future research.

### Strengths and limitations

The advantages of our study include its relatively large sample size, using 11 years of data, and its high response rates, which enables the provision of norm data and tentative cutoff scores of the JSE-S by gender among Japanese medical students. Several previous studies demonstrate that the mean scores of medical students’ empathy increases after educational programs/interventions. However, there are limited studies investigating educational effectiveness according to the level of empathy. Still, a study in the US reported that students’ empathy scores were lower in clinical years than in preclinical years, and that the decrease in empathy was smaller in students with high baseline empathy than those with low baseline empathy [40]. In contrast, our preliminary data indicate that the effectiveness of professional/educational programs in enhancing students’ empathy tends to be higher in students with moderate baseline empathy than in students with low or high baseline empathy. Thus, determining norm data and cutoff scores would allow us to evaluate educational effects and design educational programs and methods according to the levels of students’ empathy.

Our study has some limitations as well. First, the data were collected at a single institution, which could affect the generalizability of the findings. However, the medical school of the university in this study is typical of national medical schools in Japan in terms of the mean age at matriculation and the gender distribution of matriculants. The proportion of female students in the medical school of this university and that of all national medical schools in Japan between 2011 and 2021 was approximately the same: 31.3 and 33.2%, respectively [41]. When applying to this medical school, students face high levels of competition relative to other medical schools in Japan, but these are not extremely high. Of all 82 medical schools in Japan (national, public, and private), Okayama University is typically ranked in the top 15. Alongside their preparatory studies for the entrance examination, many students also have experience in extra-curricular activities, such as club and volunteer activities. Therefore, the data of this study can be considered representative of all national medical schools in Japan.

Second, we were only able to compare our results with two previous studies, which, like ours, investigated the cutoff scores of first-year medical students and separated them by gender. We could not include any other studies due to the unavailability of comparable measures.

Third, we were not able to include the students’ age and experience as variables in the analyses due to the unavailability of these data. Empathy may be influenced by students’ age and experience before entering medical school. However, as most Japanese students enter medical school immediately after or within a few years of completing high school, we believe that these variables would not substantially influence the results.

Forth, in a future study, we need to confirm the validity and practicality of the cutoff scores reported in this study by comparing high-scoring (above the top JSE-S cutoff score) and low-scoring (below the bottom cutoff score) students on measures of clinical competence to examine whether differences in clinical competence ratings present as expected.

## Conclusions

The present study provides empirical data from a relatively large data sample of 11 years, which can be used as proxy norm data and tentative cutoff scores for identifying the high and low empathy scores of the JSE-S among Japanese medical school matriculants. Our findings may be nationally comparable and can be used as representative data for national medical schools in Japan. The findings could also be helpful in identifying those who may need further training to enhance their empathy and locating the relative standing of a particular individual or group on the score distribution of the JSE-S.

## Data Availability

The de-identified datasets generated and/or analysed during the current study are available from the corresponding author on reasonable request.

## Abbreviations

ANOVA: Analysis of variance
JSE: Jefferson Scale of Empathy
JSE-S: JSE-medical student version
SD: Standard deviation
US: The United States
